# Combining curcumin (C3-complex, Sabinsa) with standard care FOLFOX chemotherapy in patients with inoperable colorectal cancer (CUFOX): study protocol for a randomised control trial

**DOI:** 10.1186/s13063-015-0641-1

**Published:** 2015-03-24

**Authors:** Glen RB Irving, Chinenye OO Iwuji, Bruno Morgan, David P Berry, William P Steward, Anne Thomas, Karen Brown, Lynne M Howells

**Affiliations:** Department of Cancer Studies, University of Leicester, Leicester, LE2 7LX UK; Department of Hepatobiliary Surgery, University Hospitals of Wales, Cardiff, CF14 4XW UK

**Keywords:** Curcumin, Oxaliplatin, Colorectal cancer, Metastases, FOLFOX

## Abstract

**Background:**

The need for low toxicity adjuncts to standard care chemotherapy in inoperable colorectal cancer, with potential to improve outcomes and decrease the side-effect burden, is well recognised. Addition of the low toxicity diet-derived agent, curcumin (the active ingredient of turmeric), to standard oxaliplatin-based therapy has shown promise in numerous pre-clinical studies.

**Methods/Design:**

This study is the first to combine daily oral curcumin with standard care FOLFOX-based (5-fluorouracil, folinic acid and oxaliplatin) chemotherapy in colorectal cancer patients with inoperable liver metastases: the CUFOX trial. CUFOX comprises a Phase 1 dose-escalation study (3 + 3 + 3 design) to determine an acceptable target dose of curcumin with which to safely proceed to a Phase IIa open-labelled randomised controlled trial. Thirty three participants with histological or cytological confirmation of inoperable colorectal cancer will then be randomised to oxaliplatin-based chemotherapy with the addition of daily oral curcumin at the target dose determined in Phase I, or to standard care oxaliplatin-based chemotherapy alone (recruiting at a ratio of 2:1).

**Discussion:**

Primary outcome measures will be the determination of a target dose which is both safe and tolerable for long-term administration to individuals in receipt of first-line oxaliplatin-based chemotherapy for inoperable colorectal cancer. Secondary outcome measures will include observation of any changes in neuropathic side-effects of chemotherapy, improvement to progression-free or overall survival and identification of putative efficacy biomarkers in plasma.

The results will be disseminated via presentation at national and international conferences, via publication in appropriate peer-reviewed journals and via the Cancer Research UK/Department of Health Experimental Cancer Medicine Centre Network. This trial has full ethical and institutional approval, and commenced recruitment in February 2012.

**Trial registration:**

ClinicalTrials.gov (NCT01490996, registered 7^th^ December 2011), European Drug Regulating Authorities (EudraCT 2011-002289-19, registered 13^th^ May 2011), UKCRN ID#10672.

## Background

### Background and rationale

Colorectal cancer (CRC) is the fourth most common cancer worldwide. It carries the second highest cancer-related mortality in Western countries with a lifetime incidence of ~1 in 15 [1,2]. Up to a fifth of CRC patients will present with metastatic disease, typically in the liver [3], and half of those undergoing resection for primary disease will develop metastases. The majority of these patients are not amenable to curative surgery [4] and experience a 5-year survival rate of less than 10%. The median survival for patients with un-resectable colorectal liver metastases in the absence of chemotherapy is approximately 8 months [5], compared with up to 24 months with chemotherapy in patients with KRAS (Kirsten rat sarcoma homologue) wild-type positive cancers [6,7]. Unfortunately, approximately 40% of such cancers display chemo-resistance from the outset and eventually all will fail to respond.

The mainstay of chemotherapy for CRC in most countries has been oxaliplatin-based, commonly with 5-fluorouracil (5-FU) and folinic acid, collectively known as FOLFOX. In the UK in 2002, the National Institute of Clinical Excellence first issued guidelines [8] for oxaliplatin to be combined with 5-FU/folinic acid and administered as first-line therapy. Compared with many cytotoxic regimens, FOLFOX is relatively well tolerated but dose-limiting side-effects are common (reviewed in National Institute of Clinical Excellence CG131 [8,9]). Diarrhoea, due to mucositis, can occur in up to a third of people receiving 5-FU [10], which can be life threatening. Patients may develop neutropaenia, increasing susceptibility to infection. The majority of patients (90%) will develop oxaliplatin-induced peripheral sensory neuropathy to some extent [11] and, after nine cycles, up to a third of patients will require dose reduction or cessation [12].

Strategies for overcoming chemo-resistance and/or augmenting the action of therapy are being investigated with the aim of extending the time that chemotherapy can be tolerated whilst maintaining efficacy and postponing resistance without increased drug toxicities. Naturally occurring dietary agents are attractive in this setting because they offer a potentially favourable side-effect profile with good patient tolerability. Interest in dietary agents has continued as evidence of anti-carcinogenic activity increases (reviewed in [13]). Curcumin is a derivative of the spice turmeric (*Curcuma longa*), typical to Asian cuisine. The use of turmeric in the field of medicine was described in Asia thousands of years ago (reviewed in [14]). Of note, Asian populations experience approximately an eighth of the incidence of bowel cancer than that of Western populations (30.8 versus 3.9 cases per 100,000 in the UK and India, respectively) [2] and diet is likely to be an important underlying factor.

Curcumin can interact beneficially with a wide variety of pathophysiological processes including maintenance of the cell cycle, carcinogenesis, wound healing and inflammation (reviewed in [15]). The effects of curcumin in combination with chemotherapy have been explored in a variety of cancer cell lines and *in vivo* models. There is a growing body of pre-clinical evidence repeatedly reporting reductions in tumour volume and metastatic features when curcumin is combined with chemotherapy, as compared to either agent alone [16]. We have previously reported that curcumin augments the effect of oxaliplatin against CRC cell lines, and restores efficacy in a cellular model of chemo-resistance [17]. Furthermore, these findings were reproduced using a xenograft model, where a reduction in tumour size of 53% was observed following treatment with curcumin and oxaliplatin compared to a 16% reduction following treatment with oxaliplatin alone. These pre-clinical data are compelling and have provided the rationale for clinical investigation. Presently, two trials have provided early evidence of efficacy for curcumin in patients with inflammatory bowel disease. Efficacy of curcumin in the cancer setting has not yet been effectively addressed. Clinical reports of curcumin in combination with any chemotherapy regimen are confined to three small trials (two in pancreatic cancer [18,19] and one in metastatic breast cancer patients [20]) plus a case report [21]. These were small studies, not powered for efficacy, and lacked a control arm, but have provided evidence that patients with advanced cancer receiving chemotherapy can tolerate daily curcumin for several months alongside their chemotherapy.

### Objectives

The main aims of this study are to: establish a safe and suitable dose of curcumin to administer to patients receiving up to 6 months of oxaliplatin-based chemotherapy; collect patient samples for biomarker and pharmacokinetic analysis; record patient-reported outcomes, including quality of life and peripheral neuropathy scores; observe progression-free survival (PFS) and overall survival (OS).

### Trial design

CUFOX (curcumin plus FOLFOX) is a Phase I dose-escalation study rolling in to a Phase IIa randomised controlled trial combining daily oral curcumin (C3-complex; Sabinsa Corp., UT, USA) with standard care FOLFOX-based chemotherapy in patients with histological diagnosis of metastatic CRC and disease measurable by response evaluation criteria for solid tumours (RECIST) 1.1, attending oncology clinics prior to chemotherapy. The study will not incorporate a placebo-controlled arm due to additional expense of sourcing an appropriate placebo. Identification of a suitable placebo is also complicated by the issue that curcumin (E100) is highly coloured and widely used as a food colouring. For Phase IIa, participants will therefore be randomised to FOLFOX + curcumin or FOLFOX only at a ratio of 2:1.

## Methods/Design

### Study setting

This study will recruit adults referred to the University Hospitals of Leicester NHS Trust Oncology Department. The study is sponsored by the University of Leicester and will be undertaken at the University Hospitals of Leicester NHS trust UK, as a single centre study.

### Eligibility criteria

Inclusion criteria are: histological or cytological diagnosis of metastatic CRC; inoperable colorectal liver metastases; disease measurable by RECIST 1.1; adequate haematological, hepatic and renal function; age ≥18 years; Eastern Cooperative Oncology Group performance status 0 or 1; recovered from effects of any recent major surgery; post-menopausal or willing to use contraception if applicable; informed consent; life expectancy estimated to be more than 12 weeks.

The exclusion criteria include: contraindications to FOLFOX chemotherapy; peripheral neuropathy (National Cancer Institute Common Terminology Criteria >1); liver failure, uncontrolled coronary heart disease, myocardial infarction within the previous 6 months; unwilling or unable to comply with the study protocol; pregnant or lactating or contemplating pregnancy; undergone chemotherapy (other than adjuvant for CRC) or participating in another drug study; previous cancer <5 years (other than colorectal, basal cell carcinoma, *in****-****situ* cervical cancer); major surgery within 4 weeks of starting the study; potentially operable liver disease without prior chemotherapy; bone marrow depletion; co-existing active infection or serious concurrent medical condition; significant cardiovascular disease; severe valvular heart disease; congestive heart failure requiring therapy (New York Heart Association grade ≥2); bone metastases; brain or leptomeningeal metastases; surgery or hospital admissions for symptomatic intra-abdominal adhesions; colitis.

### Intervention

#### The investigational medicinal product: curcumin

C3-complex curcumin is to be obtained in a single batch with a shelf life of 3 years (Sabinsa Corp.). The raw powder consists of 79.85% curcumin and 20.15% curcuminoids, and will be encapsulated in clear “00” capsules to Good Manufacturing Practice standards by Novalabs (Wigston, Leicester, UK). Approval for release will be provided by a registered Qualified Person in accordance with Medicines and Healthcare Product Regulatory Authority standards. Each capsule will contain 500 mg C3-complex with the addition of 5% (25 mg) magnesium stearate lubricant to assist the semi-automated filling process (capsule net weight 525 mg).

Curcumin will be given orally at 0.5, 1.0 or 2 g per day. There will be a loading period of 1 week prior to commencement of chemotherapy. Curcumin will be taken daily at the specified dose throughout the course of chemotherapy. Once chemotherapy ceases, participants will be deemed ‘off trial’ and curcumin intervention will also cease.

### Modifications and cessation for gastrointestinal disturbances

Curcumin can produce diarrhoea in up to a third of patients. This is often mild and self-limiting. However, up to a quarter of patients can develop grade 3/4 diarrhoea due to 5-FU. Persisting grade 2 diarrhoea as a result of curcumin prior to receiving chemotherapy will require cessation or reduction of curcumin. If diarrhoea occurs after chemotherapy commences, 5-FU modification will be in keeping with local guidelines. Modification of curcumin dose will be as per the guidance described in Table 1.

**Table 1:** **Definitions of diarrhoea and subsequent management according to the timing of symptoms in relation to administration of study drugs**

	**Diarrhoea grade**			
**Definition/timing**	**1**	**2**	**3**	**4**
Definition	Increase <4 stools/day over baseline; mild increase in ostomy output	Increase 4–6 stools/day over baseline; moderate increase in ostomy output	Increase ≥7 stools/day; incontinence; hospitalization indicated; severe increase in ostomy output limiting self-care	Life-threatening consequences; urgent intervention indicated
Pre-chemotherapy	Observe but continue to chemotherapy	Stop, stagger or reduce curcumin, continue chemotherapy when AE grade <2	Stop curcumin, continue with chemotherapy when AE grade <2	Stop curcumin, continue with chemotherapy when AE grade <2
During chemotherapy	Observe but continue chemotherapy	Reduce or stagger curcumin	Stop curcumin, continue or reduce chemotherapy as per protocol	Stop curcumin, continue reduced chemotherapy as per protocol
48 hours after one curcumin dose reduction	Observe but continue chemotherapy	Stop curcumin, continue or reduce chemotherapy as per protocol	Stop curcumin, continue or reduce chemotherapy as per protocol	Stop curcumin, continue or reduce chemotherapy as per protocol

Symptoms were defined according to the Common Terminology Criteria for Adverse Events (Version 4). AE, adverse event.

### Adherence

Adherence will be monitored via means of a daily capsule and symptom diary over the first 4 weeks, combined with monitoring of capsule return over the study duration.

### Concomitant chemotherapy (Phase I)/control group (Phase II)

Participants in Phase I, or those randomised to the control group, will receive standard care chemotherapeutic intervention. Systemic FOLFOX-based chemotherapy will be administered every 2 weeks for up to 12 cycles via central venous catheter (body surface area capped at 2 m^2^). The scheduling will be as follows: Hour 0: oxaliplatin 85 mg/m^2^ in 250 mL glucose 5%, 2-hour intravenous (IV) infusion; folinic acid 350 mg in 250 mL glucose 5%, 2-hour IV infusion. Hour +2: 5-FU bolus 400 mg/m^2^, IV bolus; 5-FU continuous infusion 2,400 mg/m^2^, 46-hour continuous IV infusion. Chemotherapy will be delayed (or reduced) until all of the following conditions are met: neutrophils >1 × 10^9^/L, platelets >75 × 10^9^/L, full recovery from stomatitis, National Cancer Institute Common Terminology Criteria for Adverse Events grade ≤2 diarrhoea, skin or other toxicity. Modifications to FOLFOX will be in line with local procedures, and will be modified or discontinued for one of the following reasons:

1. Objective evidence of tumour progression at any site as determined by computed tomography/magnetic resonance imaging scan and/or X-ray and/or ultrasound and/or clinical examination.
2. Participant request.
3. Unacceptable toxicity as determined by objective evidence, clinical judgment or patient request.
4. Alteration of national or local policy or the availability of other potentially beneficial agents to be used alongside FOLFOX (for example, bevacizumab).
5. In the event of central line removal, infusional therapy may be switched to capecitabine, and back again if the line is re-inserted.

Any other treatment considered necessary for the patients’ safety and well-being may be given at the discretion of the investigators, and will be carried out in accordance with local practice.

### Outcomes

#### Primary endpoints

##### Safety and tolerability of FOLFOX + curcumin

Phase I: Completion of dose escalation in three consecutive participants at four capsules (or two or one if de-escalation occurs) of curcumin in combination with oxaliplatin without adverse effects attributable to curcumin 1 week after two cycles of chemotherapy.

Phase IIa: The completion of 12 cycles of chemotherapy (including dose reductions) or withdrawal from therapy by the target population.

#### Secondary endpoints

Potential for improvement to PFS, OS and neurotoxicity scores in FOLFOX + curcumin versus FOLFOX only group.

Analysis of plasma for potential biomarkers of improved efficacy and to assess levels of curcumin and its metabolites.

Analysis of lymphocyte DNA to assess whether curcumin affects platinating abilities of oxaliplatin.

### Participant timeline

The timeline of proposed interventions for this study can be found in Table 2.

**Table 2:** **Schedule of tests and procedures**

**Observation**	**Screening pre-curcumin**	**Week 1/curcumin loading**	**Week 3/before 2** ^**nd**^ **cycle**	**Week 5/before 3** ^**rd**^ **cycle**	**Week 24/after cycle 12 or withdrawal**
Informed consent^1^	X				
EORTC QLQ-C30	X				X
Curcumin questionnaire	X				X
Medical history	X				
Physical examination	X				
Weight, temperature, blood pressure, pulse	X		X	X	X
ECOG performance status (Appendix 2)	X				X
12-lead electrocardiogram	X				
Haematology, liver function, renal function^2^	X	Performed as routine prior to each cycle of FOLFOX			
Urine sample (pregnancy test for female patients)	X				
Tumour assessment^3^	X	3-monthly CT scan to 24 months.			
		6-monthly CT 24 to 48 months when appropriate			
Serum CEA^2^	X	3-monthly CEA until CT scans end			
Symptom diary	X	Daily for first 4 weeks			
Neurotoxicity questionnaire	X	Questionnaire every 2 cycles, after cycle 12 or withdrawal			
Blood samples for curcumin, platinum and biomarker analysis	X	X	X	X	X^4^
FOLFOX treatment^5^		Up to 12 cycles, at 2 week intervals			
Survival		Continuously monitored once protocol therapy has ended^6^			

^1^Can be obtained at any point prior to start of trial. ^2^Patients on folinic acid/5-fluorouracil/oxaliplatin (FOLFOX) chemotherapy will routinely have these blood tests done prior to each cycle. ^3^If no radiological assessment of disease (computed tomography (CT)/magnetic resonance imaging (MRI) of chest, abdomen and pelvis) has been done within 28 days of screening, a trial baseline scan (CT/MRI of chest, abdomen and pelvis) must be completed within 28 days of the patient’s first cycle of chemotherapy. CT scans should be repeated every six cycles (12 weeks) during FOLFOX and then 3-monthly to 24 months and 6-monthly to 48 months. ^4^Research samples after final cycle should be as close to 14 days as possible, but more than 7 and can be arranged to coincide with the next clinic appointment. ^5^FOLFOX will be up to 12 cycles (approximately 24 weeks). Central line is placed prior to chemotherapy by trained staff. ^6^Direct patient involvement in the trial will cease after curcumin has been completed. Patient episodes following this will be confined to follow-up CT scans. Patients will remain in the standard care pathway. CEA, chorioembryonic antigen; ECOG, Eastern Cooperative Oncology Group; EORTC-QLQ30: European Organisation for Research and Treatment of Cancer Quality of Life Questionnaire form 30.

### Sample size

This is the first study combining FOLFOX-based chemotherapy with curcumin, and so consequently there are no published data on which to power this study. This study will provide the first efficacy data for this regimen that can be used in the powering of future studies. The sample size for the Phase I dose escalation is pre-determined by the 3 + 3 + 3 design. Phase IIa will provide further information relating to long-term safety and tolerability of this regimen, and efficacy is a secondary outcome measure. Phase I will recruit between 9 and 18 participants, dependent on the observed rate of dose-limiting toxicity. Phase II will recruit 33 participants, 22 of whom will receive FOLFOX-based chemotherapy in combination with daily oral curcumin, and 11 of whom will be randomised to the control arm receiving standard care chemotherapy.

### Recruitment

Potential participants will be identified via the weekly cancer multidisciplinary team meetings and referrals to oncology clinic. Participants who have been informed of their diagnosis and are being offered chemotherapy will be given or posted a patient information sheet (PIS). During their attendance at the oncology department they will be introduced to a trial investigator usually by the oncologist arranging the chemotherapy. The investigator will discuss the trial and PIS and answer any questions that potential participants may have. They will be given a minimum of 24 hours to consider the PIS before being invited to participate in the study. Patients can be seen at their second pre-chemotherapy clinic appointment to obtain written informed consent. Upon obtaining written informed consent from each participant, baseline evaluations can be undertaken. Baseline evaluations will include a complete physical examination (body weight, height; pulse, and assessment of Eastern Cooperative Oncology Group performance status). Body surface area must also be determined for FOLFOX dosing. Required haematological and clinical chemistry measurements will include haemoglobin, white blood cell count with differential if abnormal, platelet count, urea, creatinine, sodium, potassium, alkaline phosphatase, alanine aminotransferase, albumin, and bilirubin. Chorioembryonic antigen will be measured in all patients at baseline and, if elevated, every 12 weeks thereafter.

### Phase I traditional escalation response model (3 + 3 + 3) study

The sample size for Phase 1 is determined by the maximum dose desired (2 g/day based on previous trials within the local populace) and the number of tiers selected to step up to this dose (three tiers; 500 mg/day, 1 g/day and 2 g/day). In the absence of dose-limiting toxicity (DLT), only three patients per tier will be required (n = 9); however, the “worse-case” scenario is taken into consideration, allowing resources to repeat each tier once if necessary (n = 18).

**Figure 1:**
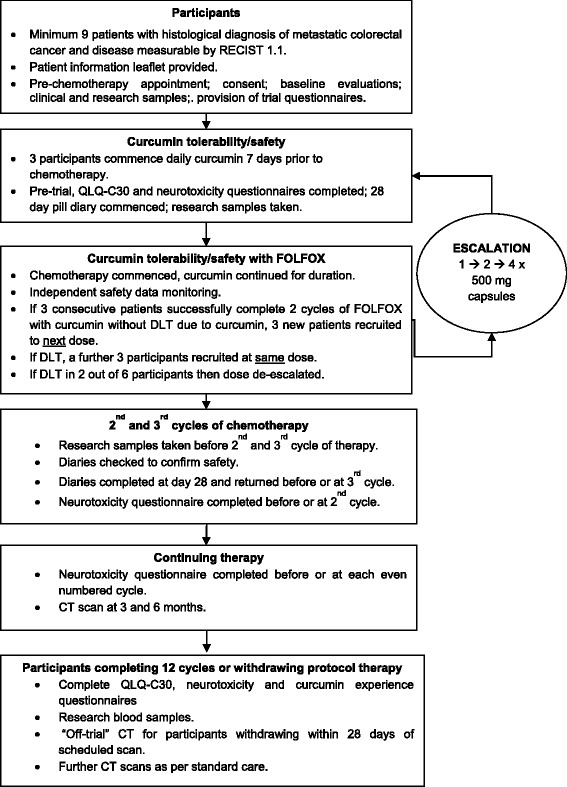
**Trial schema for Phase 1 traditional escalation response.** CT, computed tomography; DLT, dose-limiting toxicity; FOLFOX, folinic acid/5-fluorouracil/oxaliplatin; QLQ-C30, Quality of Life Questionnaire C-30; RECIST, response evaluation criteria for solid tumours.

Three patients meeting the inclusion criteria will receive one oral capsule (500 mg) per day of curcumin (Figure 1), commencing 7 days prior to chemotherapy to allow acclimatisation and any curcumin-induced adverse events (AEs) to manifest prior to chemotherapy. In the absence of curcumin-related toxicity after 1 week, patients will commence standard care FOLFOX-based chemotherapy (modified to capecitabine + oxaliplatin, or FOLFOX + bevacizumab where appropriate). After a 4-week enhanced early monitoring phase, and in the absence of curcumin-associated dose-limiting toxicity (DLT) in three consecutive patients, a further three participants will be recruited at 1 g/day oral curcumin (two capsules), and then to the maximum target dose of 2 g/day (four capsules). In the event of a DLT attributable to curcumin, the participant will cease involvement in the trial and a further three participants will be recruited at that dose level. If a participant withdraws for a reason unrelated to curcumin, only that participant will be replaced. Independent data monitoring will be convened prior to each dose escalation and prior to commencement of Phase II to ensure patient safety. De-escalation will be mandatory if more than two DLTs due to curcumin among six participants are observed within 4 weeks of commencing curcumin.

### Phase II

Phase II will be an open-labelled, two-armed, randomised controlled trial/feasibility study, consisting of a FOLFOX only (control) group (11 participants) and a FOLFOX + curcumin group (22 participants). Patients will be managed identically to those recruited to the traditional escalation response model. Primary endpoints will remain focussed on drug safety, tolerance and the feasibility of undertaking a larger scale study. A group receiving chemotherapy alone is to be included principally to provide control samples for the proposed biomarker studies, but also to generate data informing on quality of life, disease response, PFS, OS and neuropathic side-effects.

### Randomisation for Phase II

Consecutive unique identifying numbers (1 to 33) will be assigned a group via an electronic random number generator at a ratio of 2:1 (number 1 = combination therapy; number 2 = control group receiving FOLFOX-based therapy only). Groupings will be placed in a sealed envelope bearing the unique identifying number. These will be blinded to the recruiting researchers and patients until informed consent is obtained, whereupon grouping can be revealed. The randomisation list is to be kept separate to the site file and a copy held by the trial pharmacist.

### Data collection

#### Quantitative data

Clinical response and survival will be monitored as per standard care protocols. Computed tomography scans will measure disease response using RECIST 1.1 [22] until progression is observed. OS data will be obtained at the end of the trial, defined by death of the last patient recruited, from the regional cancer network database.

AE and serious adverse event (SAE) reporting will be conducted using National Cancer Institute Common Terminology Criteria AE version 4 (US Department of Health and Human Services, 2010), in accordance with both the Medicines for Human Use (Clinical Trials) regulations 2004 (SI2004/1031; SI2006/1938) and International Conference on Harmonisation Good Clinical Practice. Routine haematological and biochemical analyses will be performed as per standard care prior to each chemotherapy cycle. Expected AEs associated with curcumin are likely to be gastrointestinal in nature, consisting primarily of diarrhoea, nausea and bloating. It is currently unknown as to whether addition of curcumin to FOLFOX-based chemotherapy may increase known gastrointestinal side effects of 5-FU.

Further to collection of quantitative safety data, a variety of laboratory research measures will be assessed. Research bloods will be taken at baseline, end of week 1, prior to the 2nd and 3rd cycles of chemotherapy, and upon completion or withdrawal from the study. Briefly, curcumin and metabolites will be extracted from plasma and undergo chromatographic analysis using a high performance liquid chromatography-ultraviolet assay developed and validated in-house. Proposed biomarker studies include in-house high-definition mass spectrometry, proteomic analysis of plasma for prospective identification of markers of curcumin efficacy, and analysis of circulating tumour microRNA. In addition, quantification of platinated lymphocyte DNA will be undertaken using inductively-coupled plasma mass spectrometry to assess whether curcumin may interfere with DNA adduct formation by oxaliplatin [17,23].

### Qualitative data

A neuropathic scoring questionnaire [24] will be completed by patients every two cycles throughout protocol chemotherapy, and subjects will also record the amount and timing of curcumin doses alongside any side-effects experienced as part of the early monitoring period. A 28-day diary has been designed to support monitoring during the early monitoring period. The European Organisation for Research and Treatment of Cancer Quality of Life Questionnaire form C30 will be completed at the beginning of the trial and when participants cease protocol chemotherapy. Non-validated pre- and post-trial questionnaires relating to participant’s views on curcumin, its consumption, side-effects and the trial experience will be completed at the start and end of the curcumin course.

### Data management

Data will be abstracted from the participant’s records onto paper case report forms. Data collection and archiving will be done in accordance with appropriate standard operating procedures. Case report forms are kept in secure storage within a locked room/building and personal data is kept to a minimum. Complete patient identifiable details will be stored on a separate paper recruitment log. Transcription accuracy will be cross-checked on a quarterly basis, with audit trail and responses filed in the Trial Master Site File.

### Data analysis and statistical methods

Data will be presented in summary format. This will predominantly take the form of counts or percentages for categorical outcome data with mean/median, range and standard deviations for continuous outcome data and with 95% confidence intervals where appropriate. Additional independent samples *t*-test or unpaired non-parametric tests will be performed with *P* < 0.05 taken as significant. Estimated survival times will be calculated from clinical outcome data. Statistical comparison can be made between groups; however, this study is not primarily powered to be a biomarker or efficacy-driven study and the data generated herein is intended for the design of future clinical trials. The statistical software at the University of Leicester is SPSS/PASW (Chicago, IL, USA). Statistical analyses will be supported by the University of Leicester’s Bioinformatics and Biostatistics Hub.

### Data monitoring and auditing

An independent data monitoring committee will be convened to provide safety monitoring prior to dose escalation in Phase I. The committee will subsequently meet on a quarterly basis to review any AEs, SAEs, SUSARs (Suspected Unexpected Serious Adverse Reaction) and other safety issues arising in Phase IIa. This committee may be convened at an earlier date in the event of significant safety concerns relating to the study drug. The study will audited by an external auditor every 6 months, in keeping with Sponsor and Institutional policies.

### Ethics and dissemination

Ethical approval for this study was granted by the East Midlands (Derby 1) regional ethics committee (11/EM/0263). Clinical trials approval was obtained from the Medicines and Healthcare Products Regulatory Agency. The trial is registered at ClinicalTrials.gov (NCT01490996) and with the European Drug Regulating Authorities (EudraCT 2011-002289-19), and will be conducted in accordance with good clinical practice and the principles of the Declaration of Helsinki.

Written informed consent will be obtained by delegated members of the participant’s healthcare team. Consent will be sought from participants to allow sections of their medical records to be looked at in strict confidence by responsible individuals from the study team, the Sponsor, Research Ethics Committee, NHS Trust or from regulatory authorities where relevant.

Research findings will be reported at local levels on University Hospitals of Leicester NHS Trust and University of Leicester websites. National and international dissemination of results will take place at oncology-based conferences such as those hosted by the National Cancer Research Institute, British Association for Cancer Research, and American Society of Clinical Oncology. Furthermore, results will be disseminated in both lay and scientific summary format via the websites of major funders for this study including Hope against Cancer, The Royal College of Surgeons, the Bowel Disease Research Foundation, the Cancer Research UK Experimental Cancer Medicine Centre Network and the Cancer Research UK Leicester Centre.

## Discussion

This will be the first clinical trial to investigate outcomes from the combination of oral curcumin with standard care oxaliplatin-based chemotherapy.

The target dose of 2 g/day oral curcumin has been chosen for three reasons. Firstly, our pilot study [25] showed that whilst compliance was excellent (92%) amongst patients receiving 2.35 g taken in five daily capsules, participant questionnaires revealed a potential reluctance to take larger doses which would necessitate either a greater number or size of capsule; secondly, AEs may increase at doses in excess of 4 g, and greater caution must be observed in the presence of chemotherapy; thirdly, the dose required to exert pharmacological effect remains unknown.

A mouse model of polyp prevention demonstrating efficacy of curcumin proposes that 1.6 g per day delivered in a single dose to humans is likely to be sufficient [26]. Curcumin has been detected in plasma of patients receiving 2 g daily oral curcumin, and high-performance liquid chromatography analysis of portal blood and hepatic tissue suggests that 3 to 4 g may be sufficient for activity in organs distal to the gut. Two grammes once a day is expected to be both a well-tolerated amount of curcumin yet also possess the potential to invoke a clinical response. At higher doses, participant compliance is likely to decrease and AEs increase [27,28].

As the side-effect profile of curcumin in combination with FOLFOX-based therapy is unknown, a dose escalation arm is mandated. In controlled trials researching curcumin as a single agent, the AE incidence has been similar for both control and treatment arms [29-32]. In the study by He and colleagues, which recruited patients with CRC in to a treatment and control group, 10/37 (27%) patients taking curcumin experienced diarrhoea compared with 8/32 (25%) in the placebo group [31]. Despite this, diarrhoea does appear to be a frequent side effect of curcumin [33-35], although AE symptoms are usually brief and mild.

The second phase of this trial will look more closely for potential differences to the side-effect profile of FOLFOX brought about by the addition of curcumin, via the inclusion of a control (FOLFOX only) arm. A further novel aspect of this study is to explore the putative anti-neuropathic properties of curcumin (extensively reported in pre-clinical models [36-38]) by use of a peripheral neuropathy scoring system.

To date, there are no studies providing evidence for clinical efficacy of the low-cost, low-toxicity diet-derived agent curcumin in the treatment of cancer. Relative risk or OS have been reported by studies involving small and heterogenous patient groups taking curcumin alone [18] or combined with chemotherapy [19,20,28], and curcumin does not appear to impact negatively on survival. These studies have reported that it is both safe and feasible to combine curcumin with chemotherapy for several months in cancer patients, but that larger randomised control trials are required to investigate the efficacy of curcumin-chemotherapy regimens. CUFOX is the first randomised controlled trial of its kind and has the potential to provide early evidence of clinical efficacy of curcumin within the chemotherapeutic setting.

## Trial status

At the time of article submission, the CUFOX trial has recruited 18 of 33 participants to the Phase 2 randomised controlled trial component of the study.

## Abbreviations

5-FU: 5-fluorouracil
AE: adverse event
CRC: colorectal cancer
CUFOX: curcumin plus FOLFOX
DLT: dose-limiting toxicity
FOLFOX: folinic acid/5-fluorouracil/oxaliplatin
IV: intravenous
OS: overall survival
PFS: progression-free survival
PIS: patient information sheet
RECIST: response evaluation criteria for solid tumours
SAE: serious adverse event

## References

[1] CRUK. 2010. http://www.cancerresearchuk.org/about-cancer/type/bowel-cancer/ Accessed on 12/01/15.

[2] WHO. Cancer Incidence and Mortality Worldwide in 2008. 2008. GLOBOCAN. http://globocan.iarc.fr/ Accessed on 12/01/15.

[3] Jullumstro E, Lydersen S, Moller B, Dahl O, Edna TH (2009). Duration of symptoms, stage at diagnosis and relative survival in colon and rectal cancer. Eur J Cancer.

[4] Adam R, Delvart V, Pascal G, Valeanu A, Castaing D, Azoulay D (2004). Rescue surgery for unresectable colorectal liver metastases downstaged by chemotherapy: a model to predict long-term survival. Ann Surg.

[5] de Gramont A, Figer A, Seymour M, Homerin M, Hmissi A, Cassidy J (2000). Leucovorin and fluorouracil with or without oxaliplatin as first-line treatment in advanced colorectal cancer. J Clin Oncol.

[6] Van Cutsem E, Kohne CH, Lang I, Folprecht G, Nowacki MP, Cascinu S (2011). Cetuximab plus irinotecan, fluorouracil, and leucovorin as first-line treatment for metastatic colorectal cancer: updated analysis of overall survival according to tumor KRAS and BRAF mutation status. J Clin Oncol.

[7] Primrose J, Falk S, Finch-Jones M, Valle J, O’Reilly D, Siriwardena A (2014). Systemic chemotherapy with or without cetuximab in patients with resectable colorectal liver metastasis: the New EPOC randomised controlled trial. Lancet Oncol.

[8] NICE. Improving outcomes in colorectal cancer. The clinical effectiveness and cost effectivneness of irinotecan, oxaliplatin and ralitrexed for colorectal cancer. technical appraisal 33. 2002. http://www.nice.org.uk/guidance/ta33/resources/guidance-the-clinical-effectiveness-and-cost-effectiveness-of-irinotecan-oxaliplatin-and-raltitrexed-for-colorectal-cancer-pdf.

[9] NICE. Colorectal cancer: the diagnosis and management. Clinical Guideline 131. 2014. http://www.nice.org.uk/guidance/cg131/evidence/cg131-colorectal-cancer-full-guideline2. Accessed 15/01/15.

[10] Levy E, Piedbois P, Buyse M, Pignon JP, Rougier P, Meta-Analysis Group Inc (1998). Toxicity of fluorouracil in patients with advanced colorectal cancer: effect of administration schedule and prognostic factors. J Clin Oncol.

[11] Argyriou AA, Cavaletti G, Briani C, Velasco R, Bruna J, Campagnolo M (2013). Clinical pattern and associations of oxaliplatin acute neurotoxicity: a prospective study in 170 patients with colorectal cancer. Cancer.

[12] Leonard GD, Wright MA, Quinn MG, Fioravanti S, Harold N, Schuler B (2005). Survey of oxaliplatin-associated neurotoxicity using an interview-based questionnaire in patients with metastatic colorectal cancer. BMC Cancer.

[13] Manson MM, Foreman BE, Howells LM, Moiseeva EP (2007). Determining the efficacy of dietary phytochemicals in cancer prevention. Biochem Soc Trans.

[14] Aggarwal BB, Sundaram C, Malani N, Ichikawa H (2007). Curcumin: the Indian solid gold. Adv Exp Med Biol.

[15] Irving GR, Karmokar A, Berry DP, Brown K, Steward WP (2011). Curcumin: the potential for efficacy in gastrointestinal diseases. Best Pract Res Clin Gastroenterol.

[16] Tharakan ST, Inamoto T, Sung B, Aggarwal BB, Kamat AM (2010). Curcumin potentiates the antitumor effects of gemcitabine in an orthotopic model of human bladder cancer through suppression of proliferative and angiogenic biomarkers. Biochem Pharmacol.

[17] Howells LM, Sale S, Sriramareddy SN, Irving GR, Jones DJ, Ottley CJ (2011). Curcumin ameliorates oxaliplatin-induced chemoresistance in HCT116 colorectal cancer cells in vitro and in vivo. Int J Cancer.

[18] Dhillon N, Aggarwal BB, Newman RA, Wolff RA, Kunnumakkara AB, Abbruzzese JL (2008). Phase II trial of curcumin in patients with advanced pancreatic cancer. Clin Cancer Res.

[19] Kanai M, Yoshimura K, Asada M, Imaizumi A, Suzuki C, Matsumoto S (2011). A phase I/II study of gemcitabine-based chemotherapy plus curcumin for patients with gemcitabine-resistant pancreatic cancer. Cancer Chemother Pharmacol.

[20] Bayet-Robert M, Kwiatkowski F, Leheurteur M, Gachon F, Planchat E, Abrial C (2010). Phase I dose escalation trial of docetaxel plus curcumin in patients with advanced and metastatic breast cancer. Cancer Biol Ther.

[21] Braumann C, Guenther N, Loeffler LM, Dubiel W (2009). Liver metastases after colonic carcinoma–palliative chemotherapy plus curcumin. Int J Colorectal Dis.

[22] Eisenhauer EA, Therasse P, Bogaerts J, Schwartz LH, Sargent D, Ford R (2009). New response evaluation criteria in solid tumours: revised RECIST guideline (version 1.1). Eur J Cancer.

[23] Pieck AC, Drescher A, Wiesmann KG, Messerschmidt J, Weber G, Strumberg D (2008). Oxaliplatin-DNA adduct formation in white blood cells of cancer patients. Br J Cancer.

[24] Almadrones L, McGuire DB, Walczak JR, Florio CM, Tian C (2004). Psychometric evaluation of two scales assessing functional status and peripheral neuropathy associated with chemotherapy for ovarian cancer: a gynecologic oncology group study. Oncol Nurs Forum.

[25] Irving GR, Howells LM, Sale S, Kralj-Hans I, Atkin WS, Clark SK (2013). Prolonged biologically active colonic tissue levels of curcumin achieved after oral administration - a clinical pilot study including assessment of patient acceptability. Cancer Prev Res (Phila).

[26] Perkins S, Verschoyle RD, Hill K, Parveen I, Threadgill MD, Sharma RA (2002). Chemopreventive efficacy and pharmacokinetics of curcumin in the min/+ mouse, a model of familial adenomatous polyposis. Cancer Epidemiol Biomarkers Prev.

[27] Cheng AL, Hsu CH, Lin JK, Hsu MM, Ho YF, Shen TS (2001). Phase I clinical trial of curcumin, a chemopreventive agent, in patients with high-risk or pre-malignant lesions. Anticancer Res.

[28] Epelbaum R, Schaffer M, Vizel B, Badmaev V, Bar-Sela G (2010). Curcumin and gemcitabine in patients with advanced pancreatic cancer. Nutr Cancer.

[29] Baum L, Cheung SK, Mok VC, Lam LC, Leung VP, Hui E (2007). Curcumin effects on blood lipid profile in a 6-month human study. Pharmacol Res.

[30] Baum L, Lam CW, Cheung SK, Kwok T, Lui V, Tsoh J (2008). Six-month randomized, placebo-controlled, double-blind, pilot clinical trial of curcumin in patients with Alzheimer disease. J Clin Psychopharmacol.

[31] He ZY, Shi CB, Wen H, Li FL, Wang BL, Wang J (2011). Upregulation of p53 expression in patients with colorectal cancer by administration of curcumin. Cancer Invest.

[32] Ide H, Tokiwa S, Sakamaki K, Nishio K, Isotani S, Muto S (2010). Combined inhibitory effects of soy isoflavones and curcumin on the production of prostate-specific antigen. Prostate.

[33] Lao CD, Ruffin MT, Normolle D, Heath DD, Murray SI, Bailey JM (2006). Dose escalation of a curcuminoid formulation. BMC Complement Altern Med.

[34] Sharma RA, McLelland HR, Hill KA, Ireson CR, Euden SA, Manson MM (2001). Pharmacodynamic and pharmacokinetic study of oral Curcuma extract in patients with colorectal cancer. Clin Cancer Res.

[35] Vareed SK, Kakarala M, Ruffin MT, Crowell JA, Normolle DP, Djuric Z (2008). Pharmacokinetics of curcumin conjugate metabolites in healthy human subjects. Cancer Epidemiol Biomarkers Prev.

[36] Babu A, Prasanth KG, Balaji B (2014). Effect of curcumin in mice model of vincristine-induced neuropathy. Pharm Biol.

[37] Joshi RP, Negi G, Kumar A, Pawar YB, Munjal B, Bansal AK (2013). SNEDDS curcumin formulation leads to enhanced protection from pain and functional deficits associated with diabetic neuropathy: an insight into its mechanism for neuroprotection. Nanomedicine.

[38] Zhao WC, Zhang B, Liao MJ, Zhang WX, He WY, Wang HB (2014). Curcumin ameliorated diabetic neuropathy partially by inhibition of NADPH oxidase mediating oxidative stress in the spinal cord. Neurosci Lett.

